# Spontaneous Enteral Migration of a Feeding Jejunostomy Tube: An Unusual Complication

**DOI:** 10.7759/cureus.34861

**Published:** 2023-02-11

**Authors:** Ashikesh Kundal, Sudhir Singh, Jyoti Sharma, Dhivakar S, Summi Karn

**Affiliations:** 1 Department of General Surgery, All India Institute of Medical Sciences, Rishikesh, Rishikesh, IND

**Keywords:** nutrition, postoperative complications, feeding tube migration, feeding jejunostomy, acute intestinal obstruction

## Abstract

Established consensus suggests that enteral nutrition is more beneficial in patients with a functioning gut than parenteral nutrition. It helps in early physical rehabilitation from a disease or surgical stress and is associated with fewer complications compared to parenteral nutrition. Jejunal feeding is one of the routine modes of enteral nutrition in patients with gastric dysfunction, either due to surgery or critical illness. Various complications have been reported when using feeding tubes, grouped as mechanical, infectious, gastrointestinal, and metabolic. Here, we report an unusual case of a 47-year male with a history of prepyloric perforation repair leak who presented to us on postoperative day 14 with an enterocutaneous fistula and a feeding jejunostomy tube in situ. He was evaluated and managed conservatively and discharged on enteral feeds, both orally and via a jejunostomy tube. One month after discharge, he presented with features of intestinal obstruction with a missing jejunostomy tube. Radiological investigations suggested enteral migration of the jejunostomy tube, which was managed non-operatively, and the patient was discharged on day three post-admission after per rectal expulsion of the tube.

## Introduction

Early resumption of feeding is essential in managing surgical patients to counteract the surgical stress causing a catabolic state and enhance postoperative recovery, resulting in a shorter hospital stay. Enteral feeding is preferred over parenteral nutrition in patients with a functional gastrointestinal tract [1,2]. While the oral route is considered the best route for enteral nutrition, feeding through a jejunostomy tube is preferred in patients unable to feed adequately through the oral route due to pathology in the upper gastrointestinal tract, i.e., esophageal, gastric, or prepyloric pathology [3]. Jejunal tube feeding is performed as an ancillary procedure in various major upper gastrointestinal tract surgeries where gastric dysfunction is expected [4]. It is a simple, cheap, and easy way of enteral nutrition through small bowel access. However, various complications related to feeding jejunostomy (FJ) have been reported and classified as mechanical, metabolic, nutritional, and septic complications. Although tube obstruction, displacement, kinking, coiling, and bowel perforations have been commonly reported, enteric migration of FJ is a rare and dreaded occurrence [5,6]. This case report describes an unusual complication of enteric migration of the FJ tube in a patient with postoperative enterocutaneous fistula along with its management.

## Case presentation

A 47-year-old male underwent exploratory laparotomy with modified Graham’s patch repair at a private hospital for non-steroidal anti-inflammatory drug-induced prepyloric perforation. On postoperative day two, he was re-explored with suspicion of a patch repair leak. Primary repair of the perforation site with FJ was done using a 16 Fr Foley catheter. The patient developed bilious discharge from the midline wound postoperatively and was referred to our institute for further management.

On presentation, the patient had a high-output enterocutaneous fistula through the midline wound with an FJ tube in situ, which was fixed to the anterior abdominal wall with a silk suture. He had no signs of peritonitis. The patient was resuscitated and managed conservatively on the lines of an enterocutaneous fistula. He was started on antibiotics, parenteral nutrition, and enteral feeds via a jejunostomy tube with adequate skin care. A three-in-one total parenteral nutrition consisting of dextrose, amino acids, and lipid emulsion was supplemented along with curd-based jejunostomy feeds. About 50% of the total caloric requirement was supplemented by total parenteral nutrition (TPN) while the rest was supplemented by jejunostomy feeds. The fistula output gradually decreased over the course of the hospital stay. TPN was gradually tapered and jejunostomy feeds were increased. A low-residue oral diet consisting of bread, eggs, bananas, and roasted gram flour (sattu) was introduced, and the patient was discharged after 10 days of admission on oral and jejunal feeds.

**Figure 1:**
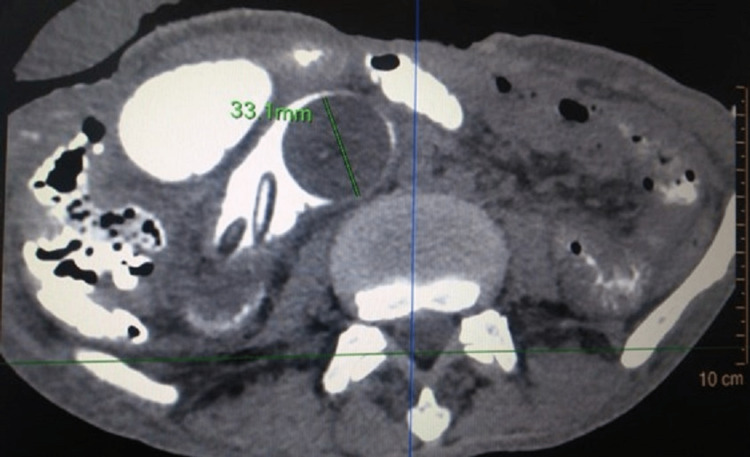
Contrast-enhanced CT showing Foley catheter bulb in the terminal ileum.

**Figure 2:**
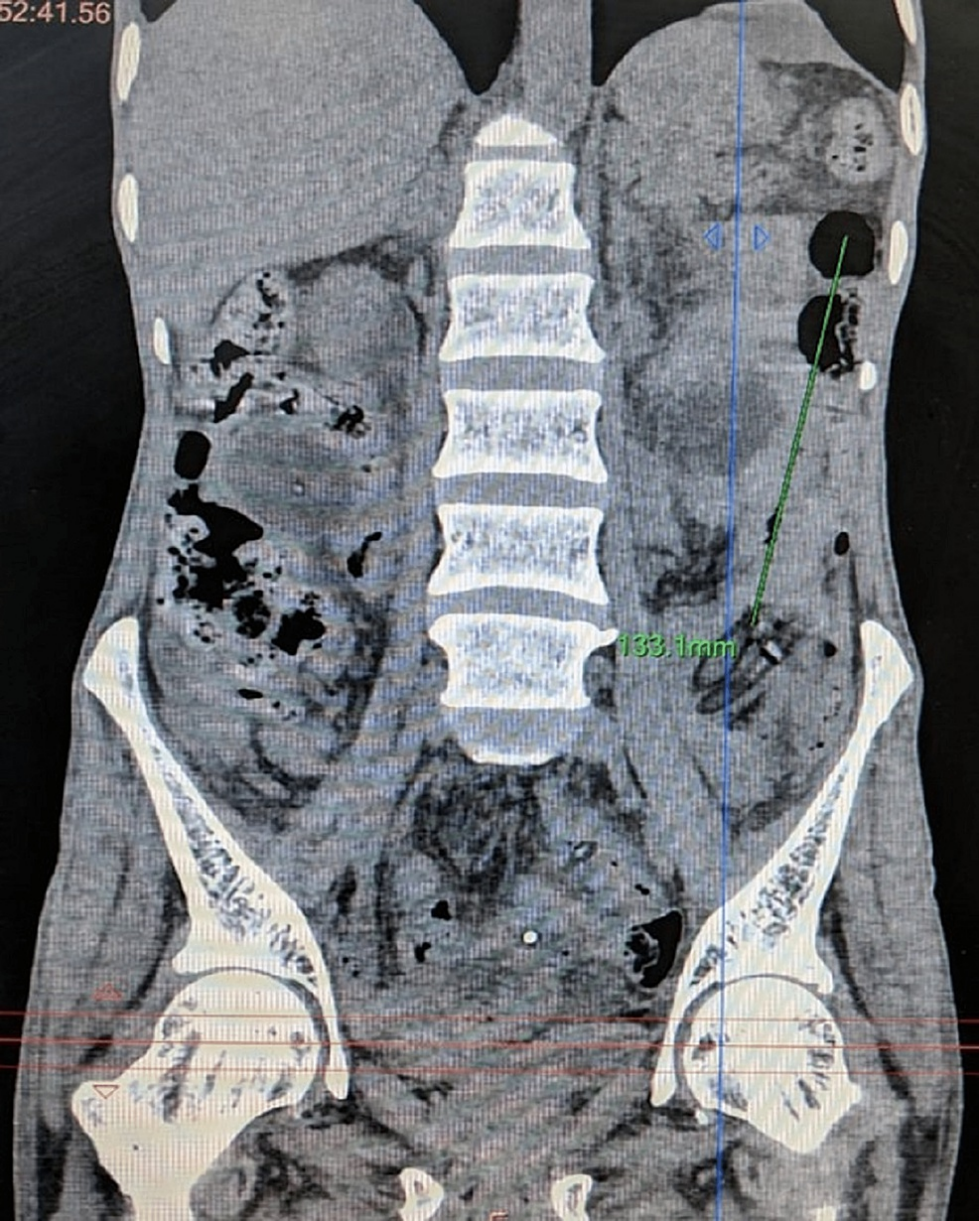
Contrast-enhanced CT (coronal section) showing a displacement of 133.1 mm of Foley catheter from the jejunotomy site.

**Figure 3:**
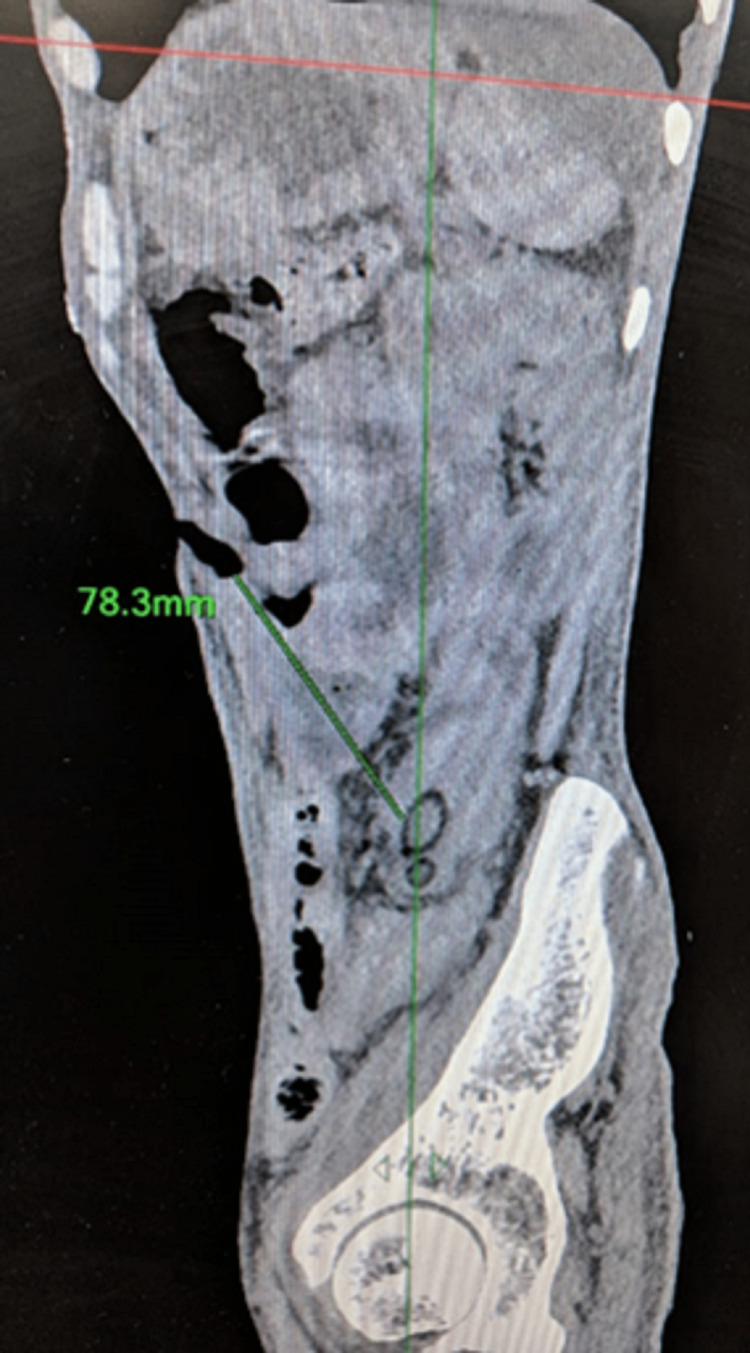
Contrast-enhanced CT (sagittal section ) showing a 78.3 mm displacement of Foley from the jejunostomy site.

The patient was advised to visit the outpatient department after 10 days but he did not follow up. He then presented to the emergency one month later with features of intestinal obstruction and the absence of the jejunostomy tube. He denied any history of accidental pulling out of the tube. On abdominal examination, the enterocutaneous fistula was replaced by a healed scar, and an opening measuring approximately 1 × 1 cm was seen at the previous FJ site. The abdomen was distended with exaggerated bowel sounds. With suspicion of antegrade migration of the FJ tube, contrast-enhanced CT (CECT) of the abdomen was done, which showed an inflated balloon of Foley’s catheter with a 33.1 mm diameter situated at a displacement of 78.3 mm in the sagittal section and 133.1 mm in the coronal section from the location of the tube jejunostomy site on the anterior abdominal wall (Figures 1-3).

**Figure 4:**
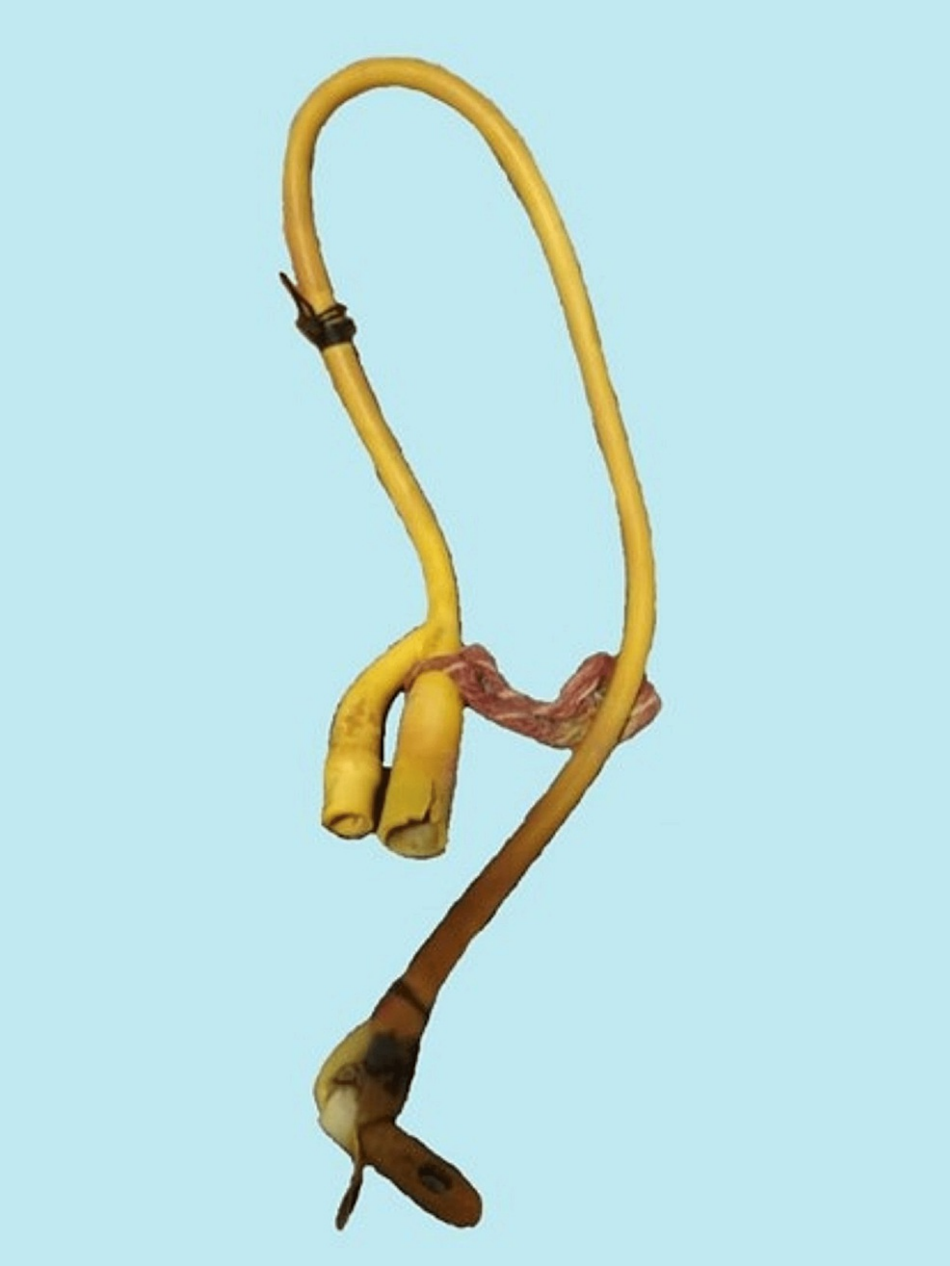
Foley catheter expelled per rectally.

Interventional radiologists performed a fluoroscopic-guided rupture of Foley’s catheter balloon, which was expelled intact, per rectum, after the third day of the procedure, and the patient was discharged (Figure 4).

On follow-up after 10 days, the patient had recovered well, and the FJ site was completely healed.

## Discussion

Enteral tube feeding is the preferred mode of nutrition in patients who cannot tolerate it orally but have a functional gastrointestinal tract. The enteral tube is placed distal to the pathologic part of the upper gastrointestinal tract [1-3]. The tube opted for FJ can be a simple Ryle’s tube, Foley’s catheter, mushroom-tip catheter, or special feeding catheter, depending on the availability and the surgeon’s preference [7,8]. Enteral tube placement has complications, as with other procedures. The incidence of complication rates mentioned in the literature is approximately 12% in patients with enteral tubes, which have been grouped under gastrointestinal, mechanical, or metabolic complications [5,6]. Common complications include bowel obstruction, bowel perforation, intussusception, or, as in our case, enteral migration.

Although a rare complication, enteral migration of the jejunostomy tube can lead to grave consequences, including bowel obstruction, pressure necrosis, and subsequent bowel perforation. Various factors must be anticipated and taken care of beforehand to prevent the migration of tubes during the follow-up visit. Adequate tube fixation is essential, and care must be taken to ensure its integrity during follow-up. This fixation may cut through and lead to migration or expulsion of the tube, as was suspected in this case. The use of retention disks and retention rings for holding the feeding tube at the fixation site is considered better for preventing migration. Patient education is another factor that can improve the outcome of enteral tube feeding therapy. The patient and caretaker must be educated on how to use and flush the tube and fixation site care and should be vigilant for signs of infection or impending migration. This will help in the early recognition and reporting of feeding tube-related complications.

The available literature has mentioned both conservative management with spontaneous per rectal expulsion of the tube and surgical removal of the migrated jejunostomy tube for the management of such cases [9]. Conservative management was opted in this case, and radiological intervention played an important role in visualizing the overinflated balloon and its fluoroscopic-guided rupture, which facilitated the movement of Foley’s through the gut and reduced the subsequent risk of pressure necrosis and perforation. Here, gut peristalsis proved as an excellent therapeutic modality in itself. However, close monitoring after admission is essential in the case of conservative management. Frequent clinical examination is necessary to recognize possible serious consequences such as acute intestinal obstruction or perforation. Failure of conservative management demands surgical or endoscopic removal.

## Conclusions

Apart from a meticulous creation of FJ, efforts should be made to avoid enteral migration of the jejunostomy tube. These include proper fixation of the tube with the parietal wall and skin, use of an external retention device, use of tubes with a dilated external end, marking of the outer part of the tube, frequent checking of the position and outer length of the tube before each feeding, and educating patients and their caretakers to examine the fixation site for signs of suture disruption. Conservative treatment is viable for managing patients with a migrated tube but requires close clinical monitoring. Moreover, it also depends on the patient’s clinical condition and the surgeon’s discretion in choosing a suitable treatment option. If conservative management fails, surgical or endoscopic procedures must be kept as a last resort.
